# Anatomical Variation of the Superficial Palmar Branch of the Radial Artery: A Cadaveric Case Report

**DOI:** 10.7759/cureus.104407

**Published:** 2026-02-27

**Authors:** Michelle Rakaba, Olivia Ross, Aishwarya Juttu, Arunabh Bhattacharya

**Affiliations:** 1 Department of Applied Biomedical Sciences, University of the Incarnate Word School of Osteopathic Medicine, San Antonio, USA

**Keywords:** princeps pollicis artery, radial artery, radialis indicis artery, superficial palmar arch, superficial palmar branch of radial artery

## Abstract

Understanding anatomical variations in hand vasculature is critical for hand and vascular surgery. This case report describes rare unilateral variations in the course and branching of the superficial palmar branch of the radial artery (SPRA) in the hand of a 100-year-old Caucasian female donor. The forearm, wrist, and hand were dissected bilaterally to demonstrate the musculature and neurovasculature. Two anatomical variations were observed in the right hand of the donor body. The first variant was the SPRA coursing superficial to the thenar muscles after emerging from the radial artery rather than piercing or passing deep to them. The second variant was the SPRA, giving rise to both the princeps pollicis and radial indicis arteries after contributing a communicating branch to the superficial palmar arch.* *Understanding variations in the origin, course, and branching of the SPRA may be important in reducing the risk of iatrogenic injuries during wrist and hand surgeries.

## Introduction

The radial artery (RA) descends on the lateral aspect of the forearm before giving rise to the palmar carpal branch and the superficial palmar branch (SPRA) at the level of the wrist. The SPRA either pierces or passes deep to the thenar muscles before traveling medially and joins the superficial branch of the ulnar artery (UA) to form the superficial palmar arch (SPA) in the palm of the hand. The RA turns dorsally at the radial styloid process, passes deep to the tendons of the extensor pollicis brevis and abductor pollicis longus muscles, and then pierces the first dorsal interosseous muscle to enter the deep palm of the hand [1]. The artery then courses medially to anastomose with the deep branch of the ulnar artery to form the deep palmar arch (DPA), which gives rise to multiple branches, including three palmar metacarpal arteries, the princeps pollicis artery (PPA), which is the principal arterial supply to the thumb, and the radialis indicis artery (RIA), which supplies the lateral aspect of the second digit.

Studies utilizing cadaveric dissections, computed tomography (CT) angiographies, and Doppler ultrasonic flowmeter have demonstrated variations in the branching and course of SPRA, formation of the SPA, as well as in the origin of PPA and RIA [2-11]. Considerable variation exists in the formation and branches of SPA according to the classifications from Jaschtschinski, and Lippert and Pabst [12,13]. Given the importance of SPRA as the blood supply to the thenar muscles and to the SPA [1], knowledge of the variations of the SPRA in terms of its course and distribution is of vital importance in the preparation for wrist and hand surgeries. In this case report, we describe two rare unilateral anatomical variations of the SPRA in a female donor. The first variant was the SPRA coursing superficial to the thenar muscles after emerging from the radial artery rather than piercing or passing deep to them. The second variant was the SPRA, giving rise to both the PPA and RIA. Furthermore, we also discuss the embryological basis and clinical significance of these findings.

## Case presentation

**Figure 1:**
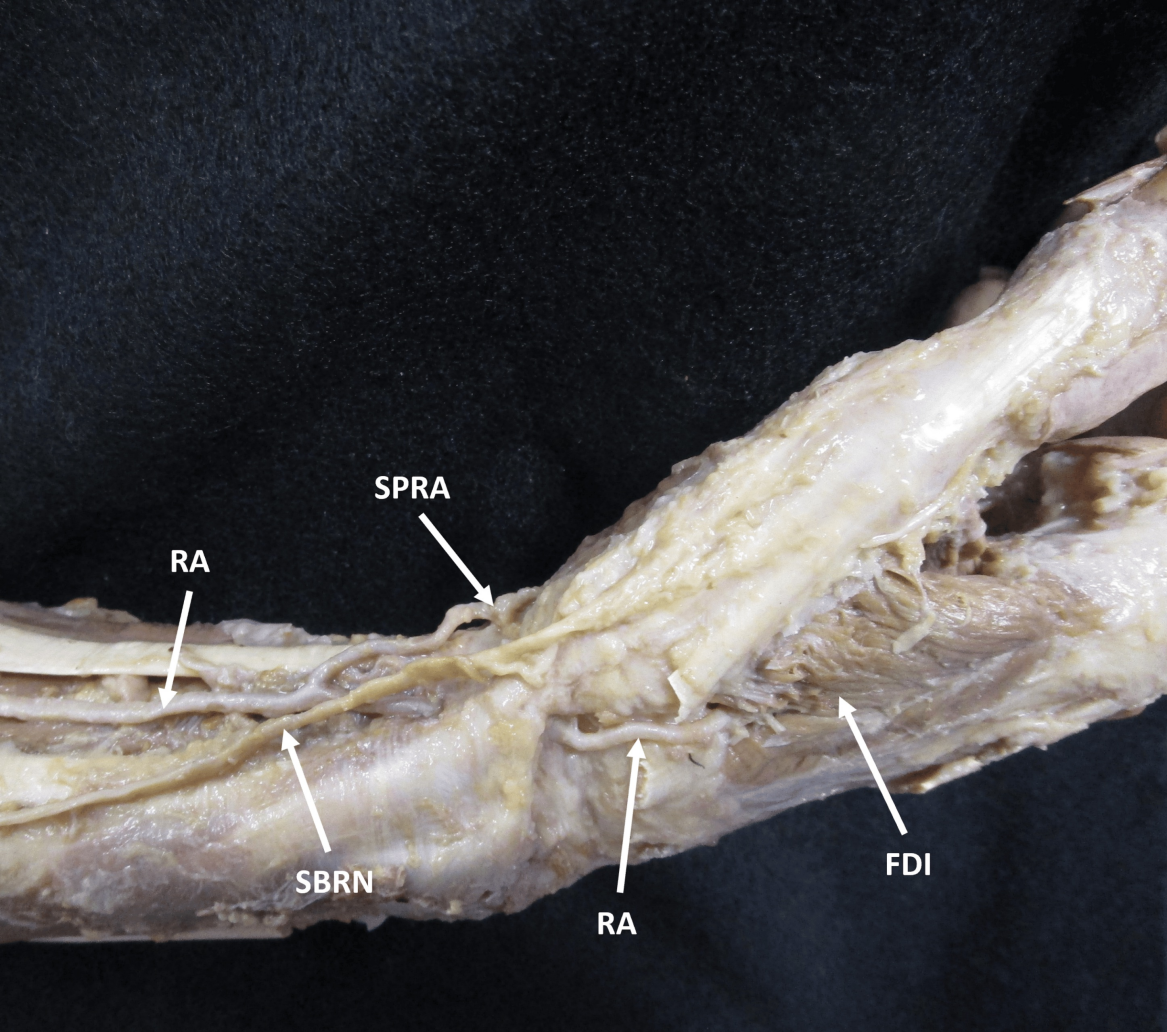
Lateral view of the right distal forearm and hand. The right side of the forearm, wrist, and hand were dissected to follow the course of the radial artery. The superficial palmar branch of the radial artery originated from the radial artery in the distal forearm. The radial artery coursed through the anatomical snuffbox before piercing the first dorsal interosseous muscle. The tendons of the extensor pollicis longus and brevis muscles were cut for better visualization of the path of the radial artery. RA: radial artery; SPRA: superficial palmar branch of radial artery; FDI: first dorsal interosseous muscle; SBRN: superficial branch of the radial nerve.

**Figure 2:**
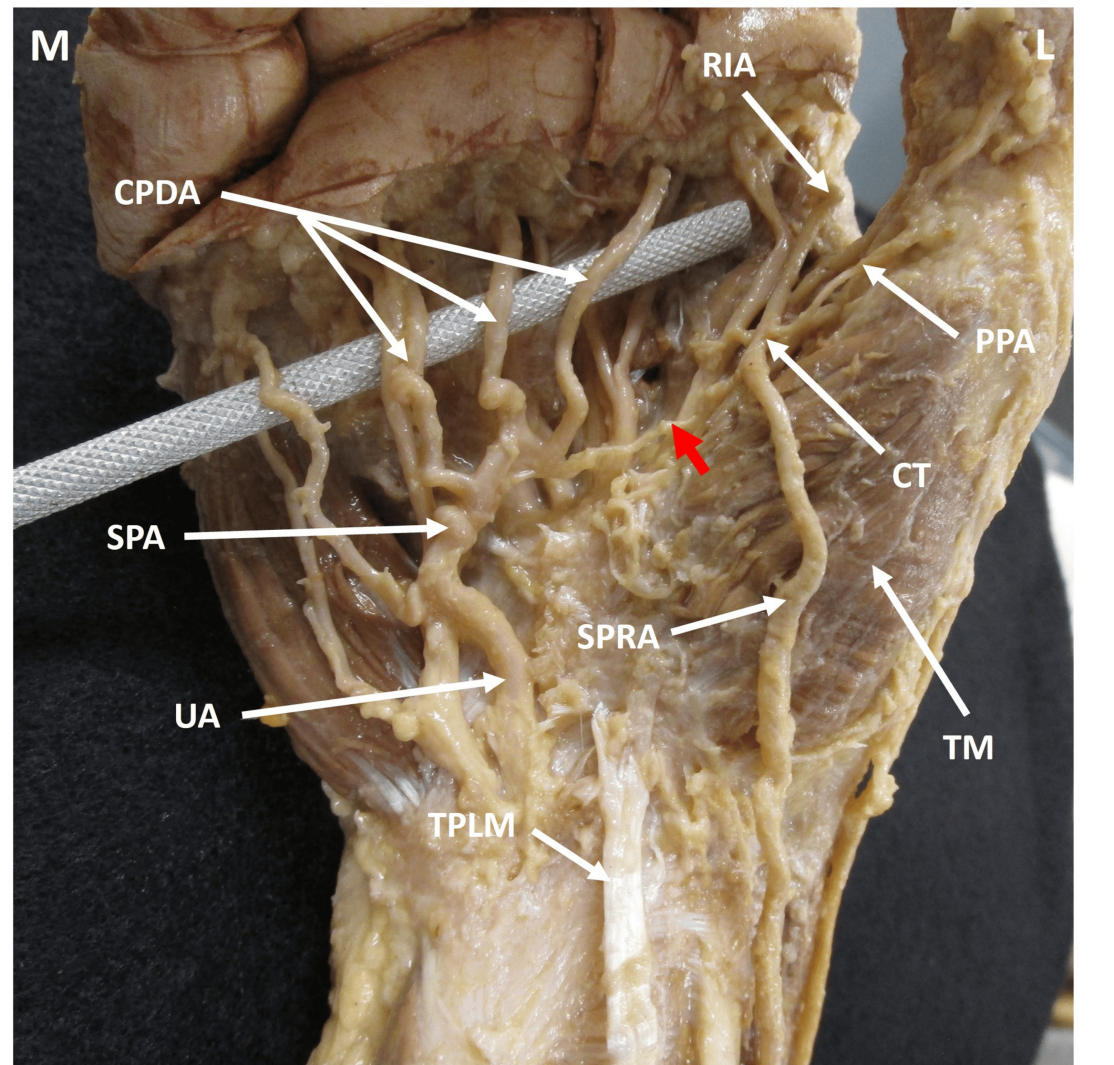
Anterior view of the right hand demonstrating the vasculature. Superficial palmar branch of the radial artery coursed superficial to the thenar muscles before providing a communicating branch (red arrow) to the superficial palmar arch. The superficial palmar branch of the radial artery provided a common trunk which gave rise to both the princeps pollicis and radialis indicis arteries. The 2nd – 4th common palmar digital arteries branched from the superficial palmar arch. CT: common trunk; SPRA: superficial palmar branch of radial artery; RIA: radialis indicis artery; PPA: princeps pollicis artery; CPDA: common palmar digital arteries; UA: ulnar artery; TM: thenar muscles; TPLM: tendon of palmaris longus muscle; SPA: superficial palmar arch; M: medial; L: lateral.

Bilateral dissection was performed on the forearm, wrist, and hand of a 100-year-old Caucasian female donor body. The donor for the study was obtained through the Willed Body Program at the University of Texas at San Antonio Long School of Medicine for the purposes of medical education and research. All donations made to the program are registered and cared for by guidelines and statutes laid out by the State of Texas. The skin, subcutaneous fat, and fascia of the forearm, wrist, and hand were removed to demonstrate the underlying musculature and neurovasculature. The SPRA originated from the RA in the distal forearm (Figure 1) before coursing superficially to the thenar muscles, instead of piercing them or passing deep to them (Figure 2). The RA continued through the anatomical snuffbox before piercing the first dorsal interosseous muscle (Figure 1). Distal to the thenar eminence, the SPRA provided a communicating branch (CB) that traveled medially across the palmar surface and formed the SPA with the superficial branch of the UA (Figure 2). The SPRA gave rise to a common trunk that branched into the PPA and RIA (Figure 2). These two variations were not noted in the cadaver’s left hand.

## Discussion

The blood supply of the hand is provided by branches from the RA and UA, especially via the SPA and DPA. SPRA normally arises from the RA before it enters the anatomical snuffbox, courses either through or deep to the thenar muscles, and joins the superficial branch of the UA to form the SPA. However, considerable variations exist in the course of SPRA as well as where it originates from the RA. In the right hand of a 70-year-old male cadaver, Kumar et al reported the course of SPRA via a ‘tunnel’ formed by the fibromuscular fibers of the thenar muscles [7]. Tagil et al showed the superficial course of the SPRA over the flexor retinaculum until it joined the UA [11]. They further reported that the SPA was solely formed by the UA in their cadaveric specimen [11]. In our case, the SPRA coursed superficially over the thenar eminence, similar to the unilateral subcutaneous course over the thenar muscles reported by Onderoglu et al. in a 52-year-old male cadaver [14]. In another study in a 70-year-old male cadaver, after passing through the anatomical snuffbox, the RA gave rise to a superficial branch that passed over the first dorsal interosseous muscle before dividing into the PPA and RIA [15]. Interestingly, in a case report involving a 17-year-old female patient, a clinical correlation was noted between the superficial course of the SPRA and repetitive pain over the thenar eminence [9], which highlights the importance of this variation. Arterial transposition of the SPRA by splitting the abductor pollicis brevis muscle led to cessation of the patient’s symptoms [9].

Once the RA enters the palm after passing through the first dorsal interosseous muscle, it anastomoses with the deep branch of the UA to form the DPA. The PPA and RIA are key branches of the radial artery that supply the thumb and the lateral side of the second digit (index finger), respectively, and typically originate from either the DPA or the RA. Some studies have reported variations in the origin of the PPA and RIA [6,8,12]. The same study by Onderoglu et al that reported the superficial course of the SPRA over the thenar muscles also showed PPA to arise from the SPRA [14]. Furthermore, the RIA originated from the SPRA at the level of the first metacarpal space before turning medially and forming the SPA with the superficial branch of the UA [14]. Lampasona et al documented the rare presence of duplicated PPA and RIA in a 90-year-old female donor [6]. The duplicated PPA and RIA in the donor body arose from the SPA [6]. In a 48-year-old male donor, Loukas et al reported a common trunk arising from the SPA, which then branched into the PPA and RIA [8]. Interestingly, the RA had no contribution to the SPA in the study and was solely formed by the UA [8]. This type of SPA is designated as an ulnar-type arch according to the classification by Jaschtschinski [12]. When a common trunk providing the PPA and RIA originates from the SPA, it is termed the first common digital artery, and the other common digital arteries from the SPA are termed the 2nd-4th common digital arteries [14]. In our donor body, the SPRA bifurcated into the PPA and RIA via a common trunk after providing a communicating branch to the SPA.

The embryological development of the SPRA is influenced by complex genetic signaling pathways that regulate vascular patterning and arterial differentiation. During early limb development, the RA forms as a branch of the brachial artery, guided by molecular signals, such as vascular endothelial growth factor (VEGF) and fibroblast growth factors (FGFs), which regulate angiogenesis and endothelial cell differentiation [16]. The formation of the SPRA specifically is influenced by genetic factors involved in forelimb vascular patterning, including Eph/ephrin signaling, which controls arterial branching and navigation [2]. Additionally, HOX genes, which are critical for proximal-distal limb patterning, may influence the location and trajectory of the RA and its superficial branches, including the SPRA. Variations in these pathways can lead to anomalies, such as an absent or aberrantly coursing SPRA, or atypical connections with the UA, which may impact hand vascularization and surgical outcomes. Similarly, the embryonic development of the PPA and RIA is coordinated through patterned outgrowths from the RA and is influenced by the same genetic pathways governing limb vasculature, including VEGF and HOX gene expression [17]. These embryological influences give rise to the diverse vascular configurations seen in the hand, underscoring the clinical importance of recognizing such variations to avoid complications during invasive procedures and surgical interventions.

The anatomical variation of the SPRA is of considerable clinical importance, particularly in the fields of hand surgery, vascular surgery, and diagnostic imaging. Normally, the SPRA arises at the level of the wrist and travels superficially to join the UA, contributing to the formation of the SPA. In some individuals, however, this branch may be absent, originate more proximally or distally, or have an unusual course, such as running deep to the flexor tendons or taking an atypical superficial trajectory within the palm [18]. A notable variation is the superficial course of the SPRA combined with the origin of the PPA and RIA from the SPRA itself. This configuration places the blood supply to the thumb and second digit at a higher risk of injury during invasive procedures or trauma, particularly when using the RA as an access point. These variations can significantly alter the blood supply to the palm and digits, which is crucial for preserving tissue viability following trauma or surgery. For instance, in reconstructive hand surgery or trauma cases involving the palm, knowledge of these variations is essential to avoid inadvertent damage to the artery, which could compromise the vascular supply and lead to ischemia [19]. In interventional cardiology, the RA is often used for transradial access during coronary artery stenting, especially in patients who are not candidates for open-heart surgery. Although this approach is associated with shorter hospital stays and fewer complications than the femoral route [3], in cases where the SPRA provides dominant supply to critical digits and has minimal anastomosis with the UA, there is an increased risk of ischemic complications following arterial puncture or cannulation [20]. Furthermore, procedures such as RA harvesting for coronary bypass grafting or forearm-based flap procedures in plastic and reconstructive surgery may result in inadequate perfusion to the hand if collateral flow through the SPA is insufficient. Coleman and Anson’s extensive anatomical study showed that only 34% of 650 cadaver hands exhibited the classic SPA formation, underscoring the prevalence of vascular variability and the need for preoperative vascular mapping [21]. Additionally, these variations may be overlooked in diagnostic imaging, leading to misinterpretation of vascular anatomy and incorrect assessments of blood flow. As such, the use of preoperative Doppler ultrasound or angiography is highly recommended in patients undergoing procedures involving the RA or palmar vasculature to identify potential anomalies and reduce the risk of iatrogenic injury. Therefore, a detailed understanding of the potential anatomical variations of the SPRA is vital for surgical planning, ensuring optimal surgical outcomes, preventing complications such as digital ischemia, and improving patient care.

## Conclusions

This case report documents two rare anatomical variations in a single donor. One involved the SPRA coursing superficial to the thenar muscles after emerging from the radial artery rather than piercing or passing deep to them. The other involved the SPRA giving rise to both the PPA and RIA after contributing a communicating branch to the superficial palmar arch. Knowledge of these rare anatomical anomalies may be beneficial for hand surgeons to reduce the risk of iatrogenic injuries to the RA and SPRA, and for the education and training of medical students and residents. Future research should focus on population-based angiographic or imaging studies to establish the prevalence of these vascular anomalies.
